# Emergency video-assisted thoracic surgery for ruptured pulmonary arteriovenous malformation-related hemothorax in a pregnant woman: a case report

**DOI:** 10.1186/s13256-018-1616-0

**Published:** 2018-03-19

**Authors:** Hu-Lin Christina Wang, Jia-Hao Zhang, Cheng-Hung How

**Affiliations:** 1 Division of Thoracic Surgery, Department of Surgery, Far Eastern Memorial Hospital, New Taipei City, Taiwan; 2 Division of Pulmonary Medicine, Department of Internal Medicine, Far Eastern Memorial Hospital, New Taipei City, Taiwan; 3 Division of Thoracic Surgery, Department of Surgery, National Taiwan University Hospital and National Taiwan University College of Medicine, Taipei, Taiwan

**Keywords:** Pulmonary arteriovenous malformations, Thoracoscopy, Video-assisted thoracoscopic surgery, Hemothorax, Emergent cesarean

## Abstract

**Background:**

Pulmonary arteriovenous malformations are rare vascular abnormalities that permit direct communication between the pulmonary artery and vein. During pregnancy, pulmonary arteriovenous malformation carries an increased risk of enlargement and rupture, which could lead to life-threatening hemothorax. This is the first case reporting successful thoracoscopic surgery for a pregnant woman with pulmonary arteriovenous malformation rupture-related hemothorax.

**Case presentation:**

We present a case of a 32-year-old pregnant Asian woman (31 weeks’ gestation) whose pulmonary arteriovenous malformation ruptured, leading to right-sided spontaneous tension hemothorax. First, an emergency cesarean section for hypovolemic shock-related fetal distress was performed to save the baby. Immediately afterwards, video-assisted thoracic surgery with the single-incision approach allowed us to successfully obtain hemostasis and eradication of abnormal vasculature by conducting wedge resection of the pulmonary arteriovenous malformation.

**Conclusions:**

Emergency thoracoscopic surgery for a pregnant woman with pulmonary arteriovenous malformation rupture-related hemothorax is safe and feasible. In contrast to transcatheter arterial embolization, video-assisted thoracic surgery could simultaneously achieve hemostasis for prevention of mortality, eradication of abnormal vasculature, and removal of massive thrombi.

## Background

Pulmonary arteriovenous malformations (PAVMs) are rare vascular abnormalities that allow direct communication between the pulmonary artery and vein, bypassing the capillaries. Most PAVMs are asymptomatic, but occasionally dyspnea may develop owing to a right-to-left shunt. Although hemoptysis and hemothorax are rare complications of PAVM, they can be life-threatening [1, 2]. Therefore, use of the appropriate treatment strategy is critical. Here we present the case of a pregnant woman with PAVM who developed spontaneous tension hemothorax that was successfully treated with minimally invasive thoracoscopic surgery.

## Case presentation

A 31 weeks pregnant 32-year-old Asian woman presented to our emergency department with the chief complaint of sudden-onset dyspnea and backache. Signs of acute respiratory distress, pallor with cold sweats, and hypotension (blood pressure 66/36 mmHg) were noted on arrival. According to her medical record, she had no history of significant systemic disease or medical events. She also denied any recent trauma.

**Fig. 1:**
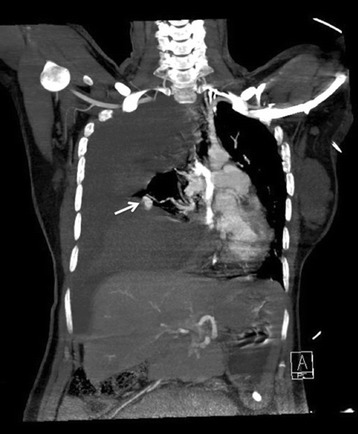
Coronal computed tomography image of the patient’s chest. A massive right-sided hemothorax and an arterial phase-enhanced lesion, compatible with a vascular anomaly (*white arrow*), in the lower lobe of the right lung

On admission, a neurological examination revealed that she was alert, with a Glasgow Coma Scale score of E4V5M6. Her cranial nerves were intact. She had no weakness, ataxia, or sensory disturbance. Her hematology results were near the normal ranges (hemoglobin 10.8 g/dl, mean corpuscular volume 92 fl, platelet count 24 × 10^3^/μl, white blood cell count, 23.37 × 10^3^/μl). Laboratory results also indicated that her renal function, liver function, and levels of serum electrolytes were normal. Hypotension was transiently responsive to fluid resuscitation. A fetal monitor showed variable decelerations, indicating fetal distress. Breath sounds over her right lung field were diminished. Bedside chest ultrasound showed massive right-sided parapneumonic effusion, and thoracentesis yielded bloody fluid. Computed tomography angiography (CTA) of her chest displayed a large right-sided hemothorax with contrast extravasation from an arterial phase-enhanced lesion in the lower lobe of her right lung and mediastinal shifting to the left (Fig. 1). Owing to the tension hemothorax, we immediately performed tube thoracostomy to relieve cardiac compression, and 2000 mL of bloody effusion was drained. Afterwards, she was placed under general anesthesia, and an emergency cesarean delivery was performed; this was immediately followed by thoracoscopic exploration to establish hemostasis.

**Fig. 2:**
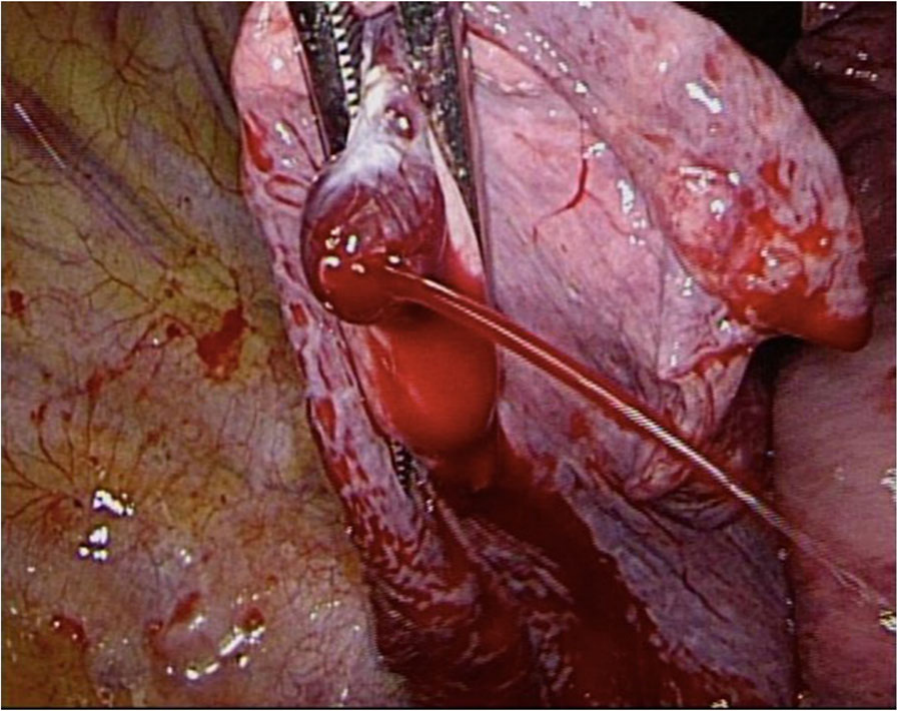
Ruptured pulmonary arteriovenous malformation in the lower lobe of the right lung showing active bleeding

**Fig. 3:**
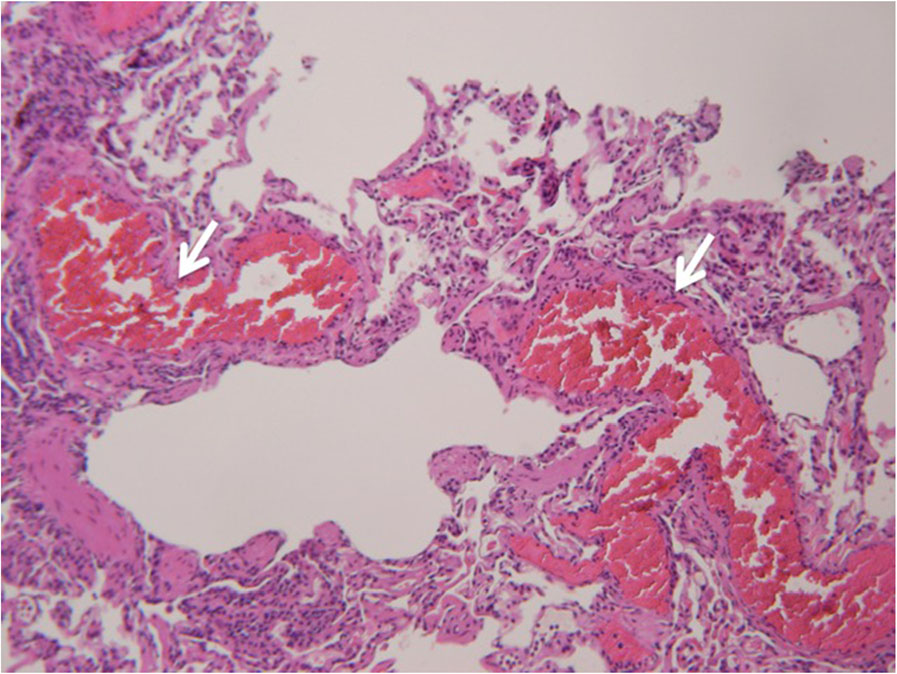
Histological images of the pulmonary arteriovenous malformations. Septa and interstitium with fresh thrombi formation (*white arrows*); hematoxylin and eosin staining, × 200 magnification

Video-assisted thoracic surgery (VATS) was performed via a single incision (5 cm in length), which was located near the mid-axillary line in the seventh intercostal space. After removal of retained thrombus inside the pleural space measuring approximately 1800 mL, a ruptured PAVM was identified in the lower lobe of her right lung (Fig. 2). After achieving hemostasis by control of the bleeding vessel with a ring clamp, her blood pressure immediately improved. Wedge resection of the lung was performed with an endostapler (ECHELON FLEX™ ENDOPATH® Staplers). Further exploration revealed a second small PAVM in the middle lobe of her right lung, which was also removed by wedge resection. Histological examination of the resected lung tissue confirmed the diagnosis of PAVM in both the lower and middle lobes of her right lung (Fig. 3).

Her postoperative recovery was uneventful. After taking analgesic medication (acetaminophen 500 mg four times a day), only mild wound pain was reported. Examinations of other organs did not identify any additional arteriovenous malformations. She was discharged 1 week later in a stable condition. She is currently healthy 18 months after surgery.

## Discussion

PAVMs are rare vascular abnormalities that can cause life-threatening hemothorax when ruptured, especially in a pregnant woman. We present a case of a 31-year-old pregnant Asian woman (31 weeks’ gestation) whose PAVM ruptured, leading to right-sided spontaneous tension hemothorax. To the best of our knowledge, this is the first case using VATS with the single-incision approach instead of transcatheter arterial embolization (TAE) to successfully obtain hemostasis and eradication of abnormal vasculature by conducting wedge resection of the PAVM.

PAVMs most commonly occur as a manifestation of hereditary telangiectasia, an autosomal dominant vascular disorder previously known as Osler–Weber–Rendu syndrome [3]. Other causes, although rare, include trauma, malignancy, hepatopulmonary syndrome, and cardiac surgery [1]. Symptoms are often due to right-to-left shunting and can include dyspnea, shunting of micro-emboli leading to ischemic strokes, and brain abscesses. Once diagnosed, evaluation for AVMs in other regions should be conducted [2].

There are two recommended therapeutic options for the treatment of patients with PAVM: TAE and surgical resection (ligature, wedge resection, segmentectomy, lobectomy, and pneumonectomy). In cases of massive hemoptysis or hemothorax, surgery is the therapy of choice [4]. TAE is a less invasive technique for the treatment of PAVM, with success rates of 85–98%; however, TAE is associated with complications including contrast nephropathy, pleuritis, paradoxical embolization, coil migration, pulmonary infarction, and transient ischemic attacks [5]. Thus, it may not be appropriate in all cases. Surgical resection of PAVM is recommended for all patients who can undergo general anesthesia; in cases of TAE failure, surgical resection of PAVM is recommended for patients with neurological complications, newborns, or central localization of PAVM [4].

Pregnant women with PAVMs are at risk of PAVM enlargement and rupture, especially in the third trimester [6]. During pregnancy, increased blood volume and cardiac output can augment blood flow through the PAVMs, potentially resulting in dilatation of the vasculature and occasionally rupture [6]. Once a PAVM ruptures, bleeding into the pleural cavity results in hemothorax and may lead to progressive dyspnea, pleuritic pain, hypoxia, and hypovolemic shock.

In two previously reported cases, pregnant women with PAVMs developed spontaneous hemothorax at the 31st [7] and 36th [8] gestational weeks. Both were successfully treated by TAE of the PAVM after cesarean deliveries.

In the present case, we performed an emergency cesarean delivery followed by thoracoscopic exploration. The use of VATS exploration allowed us to: (1) clearly identify the ruptured and bleeding PAVM and successfully achieve hemostasis by removing the PAVM via wedge resection of the lung; (2) identify another small PAVM on the surface of the lung that may not otherwise have been identified with computed tomography or angiography; and (3) remove massive thrombi intraoperatively, which greatly reduced the duration of indwelling chest drainage.

While angiography can be performed immediately after an emergency cesarean section to diagnose PAVMS and for the embolization of PAVMs, this approach may not always be ideal. First, unless a hybrid operation setting is available, it is likely that the patient will be transferred to an angiography room in an unstable hemodynamic status. Second, retained thrombi in the hemithorax may cause passive pulmonary atelectasis, which may prolong chest tube drainage. Thus, performing thoracoscopic surgery instead of angiography following the patient’s cesarean section may be preferable, since immediate and definitive hemostasis can be achieved, along with thrombi removal to provide excellent lung expansion, as was the case in our patient.

## Conclusions

Although angiography has been utilized previously to diagnose PAVMs and achieve hemostasis in pregnant women, immediate thoracoscopic surgery following emergency cesarean delivery may be a more appropriate method, as wedge resection of the PAVM to control bleeding and removal of massive thrombi can be performed simultaneously and safely.

## Abbreviations

CTA: Computed tomography angiography
PAVM: Pulmonary arteriovenous malformation
TAE: Transcatheter arterial embolization
VATS: Video-assisted thoracic surgery
